# Syntenic block overlap multiplicities with a panel of reference genomes provide a signature of ancient polyploidization events

**DOI:** 10.1186/1471-2164-16-S10-S8

**Published:** 2015-10-02

**Authors:** Chunfang Zheng, Daniella Santos Muñoz, Victor A Albert, David Sankoff

**Affiliations:** 1 Department of Mathematics and Statistics, University of Ottawa, Ottawa, K1N 6N5, Canada; 2 Department of Biology, University at Buffalo, Buffalo NY, 14260, USA

**Keywords:** whole genome duplication, angiosperms, mixture of distributions

## Abstract

**Background:**

Following whole genome duplication (WGD), there is a compact distribution of gene similarities within the genome reflecting duplicate pairs of all the genes in the genome. With time, the distribution broadens and loses volume due to variable decay of duplicate gene similarity and to the process of duplicate gene loss. If there are two WGD, the older one becomes so reduced and broad that it merges with the tail of the distributions resulting from more recent events, and it becomes difficult to distinguish them. The goal of this paper is to advance statistical methods of identifying, or at least counting, the WGD events in the lineage of a given genome.

**Methods:**

For a set of 15 angiosperm genomes, we analyze all 15 *× *14 = 210 ordered pairs of *target genome versus reference genome*, using SynMap to find syntenic blocks. We consider all sets of *B ≥ *2 syntenic blocks in the target genome that overlap in the reference genome as evidence of WGD activity in the target, whether it be one event or several. We hypothesize that in fitting an exponential function to the tail of the empirical distribution *f *(*B*) of block multiplicities, the size of the exponent will reflect the amount of WGD in the history of the target genome.

**Results:**

By amalgamating the results from all reference genomes, a range of values of SynMap parameters, and alternative cutoff points for the tail, we find a clear pattern whereby multiple-WGD core eudicots have the smallest (negative) exponents, followed by core eudicots with only the single "*γ*" triplication in their history, followed by a non-core eudicot with a single WGD, followed by the monocots, with a basal angiosperm, the WGD-free *Amborella *having the largest exponent.

**Conclusion:**

The hypothesis that the exponent of the fit to the tail of the multiplicity distribution is a signature of the amount of WGD is verified, but there is also a clear complicating factor in the monocot clade, where a history of multiple WGD is not reflected in a small exponent.

## Background

Immediately after a whole genome duplication (WGD), and for a time that is short on the evolutionary timescale, the distribution of gene similarities within the genome shows a sharp peak near 100 %, containing duplicate pairs of all the genes in the genome. With the passage of time, this peak broadens and loses volume due to the variability in the rates of decay of duplicate gene similarity and to the process of fractionation whereby one of the genes in a duplicate pair is excised or becomes unrecognizable through pseudogenization and rapid base change.

If there are two or more WGD (or higher order polyploidizations), the older peaks become so reduced and broad that they merge with the tails of the distributions resulting from more recent events, and it becomes difficult to distinguish them. The goal of this paper is to advance statistical methods of identifying, or at least counting, the WGD events in the lineage of a given genome.

The self-comparison of a genome is subject to high levels of noise due to random similarities between genes, widely shared gene domains, duplications of individual genes independent of WGD events, transposons, genome rearrangements and other factors. We can greatly attenuate this noise through recourse to procedures for detecting duplicate pairs, such as SynMap [1,2], that retain only those pairs where the two genes are in similar syntenic context, as defined by a fixed minimum number of pairs of duplicated genes not interspersed with more than a fixed number of single-copy genes.

More sensitive than genome self-comparison is the comparison of the WGD descendant *W *with another not too distantly related reference genome *R*. More orthologous genes can be detected in a *W × R *comparison than paralogs in a *W × W *analysis because fractionation does not eliminate *both *genes of a paralogous pair in *W *. Thus the orthology still shows up with the one remaining paralog in *W *and its homolog in *R*, while the paralogy is destroyed by fractionation. Indeed, we may find two long genomic regions in *W *that were originally duplicates of each other but that retain few or no duplicates between them, simply because they both contain a sufficient number of orthologs interleaved in region of *R *of comparable length. Moreover, we may find three, four or more such regions in *W *if its lineage involved more than one WGD. Thus we introduce the idea of a "superblock" as defined by two or more syntenic blocks in *W *whose corresponding blocks in *R *overlap a specified number of genes. The relationship between the number of blocks in *W *making up a superblock - its multiplicity, however, is not strictly determined by its WGD history, because of random attrition of blocks due to fractionation, disruptions due to chromosomal rearrangement, and other processes.

Motivated by the this conception of superblocks being a statistical reflection of the WGD history of a genome, we will make judicious use of SynMap and distribution-fitting in the comparison of a WGD descendant *W *with a number of reference genomes *R*1, *R*2,... in an attempt to statistically tease out the amount of polyploidization in the lineage of *W *. We find a statistic, the rate parameter *c *of an exponential fit to the tail the distribution of multiplicities, that does indeed reflect the amount of polyploidy in most of the fifteen genomes studied. Somewhat unexpectedly, however, the same statistic *c *seems to be determined in large part by the major phylogenetic grouping containing a particular genome. We are left with both empirical and theoretical questions as to the relative contribution of phylogeny and WGD history to the distribution of multiplicities.

## WGD in the flowering plants

All flowering plants (angiosperms) have WGD in their ancestry [3,4]. Two or such events are known to have preceded the angiosperm radiation, and reflect history shared with more primitive plants [5]. Additional WGD within the angiosperms have affected all known genomes except that of *Amborella trichopoda*, the descendant of the earliest diverging branch of the flowering plants [6].

For this study, we selected 15 phylogenetically diverse angiosperm genomes from the CoGe database: the basal angiosperm *Amborella trichopoda*, the moncots duckweed, sorghum and rice, the basal eudicot *Nelumbo nucifer*, and the asterids coffee, tomato, *Mimulus guttatus*, and *Utricularia gibba*, and the rosids grape, peach, cassava, poplar, *Arabidopsis thaliana *and clementine; source references available from CoGe. *Nelumbo *is known to have undergone a WGD not shared with any other sequenced species [7,8]. The monocots in our sample have all undergone three WGD, one which predates their divergence, and two each in the duckweed and cereal lineages. The asterids and rosids share a triplication event at the origin of these two large groups of plants. In addition, all the asterids except coffee have had further WGD, as have all the rosids except grape and peach.

## Duplication and fractionation, paralogy and orthology

After a WGD event, we expect 100% of the genes in a genome to be present in at least two exact copies. In this simplest model, if duplicates are lost as an exponential distribution with parameter *λ*, after a time *t* there will be *λ*^−1^*e^−λt^* duplicate pairs - paralogs - left, and 1 *− λ*^−1^*e^−λt^* single-copy genes. In contrast, between two sister genomes diverging after WGD, for each gene in the pre-WGD parent, at least one pair of orthologs should persist for a long time, since loss of both genes from a genome is likely to be lethal. There will be very many exceptions, of course, but statistically speaking we can expect far more orthologs between the sister genomes than paralogs within either one.

**Figure 1:**
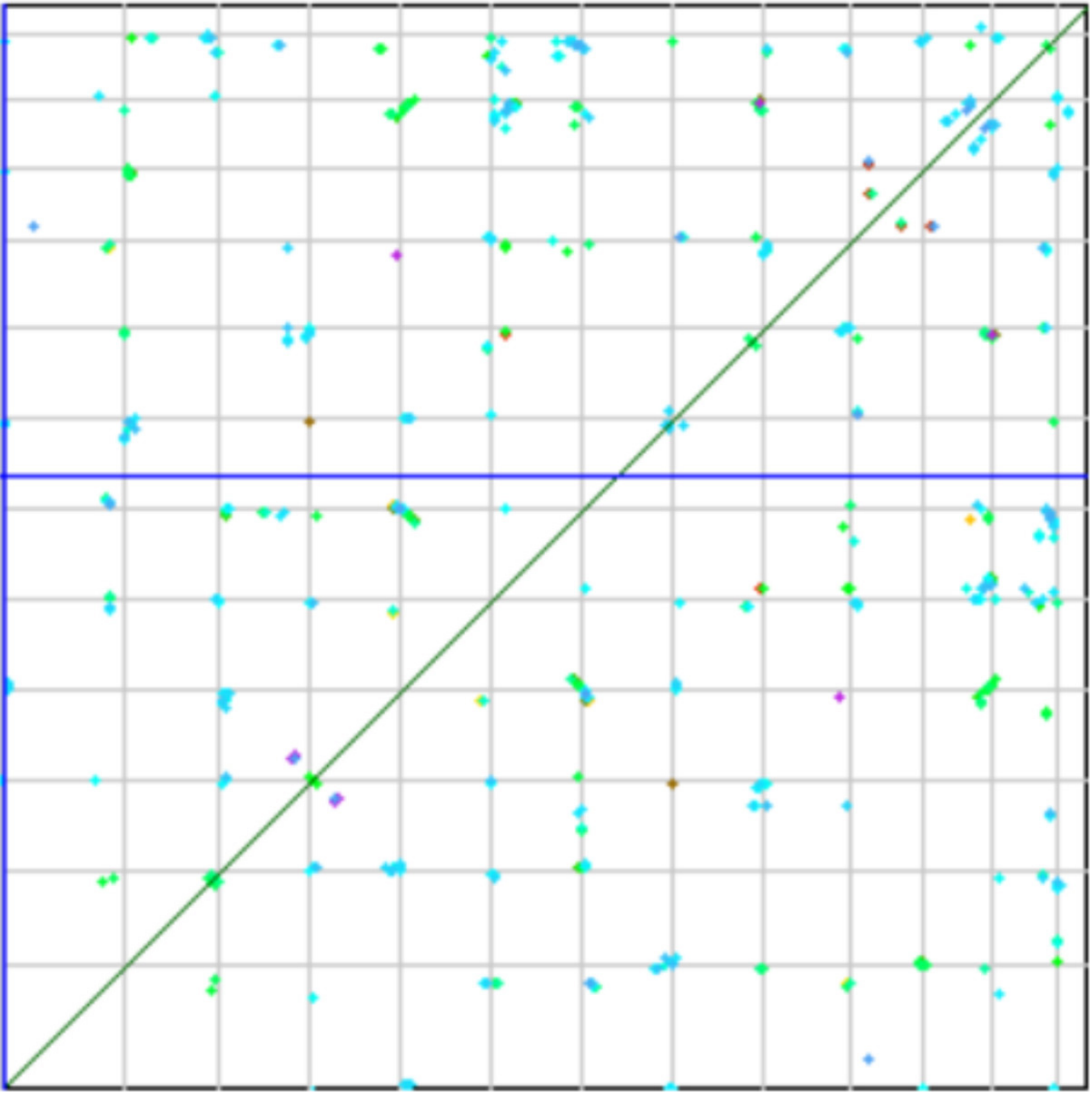
**Dot-plot of syntenic blocks in a tomato self-comparison**. Sparse distribution of syntenic blocks reflects extensive fractionation.

**Figure 2:**
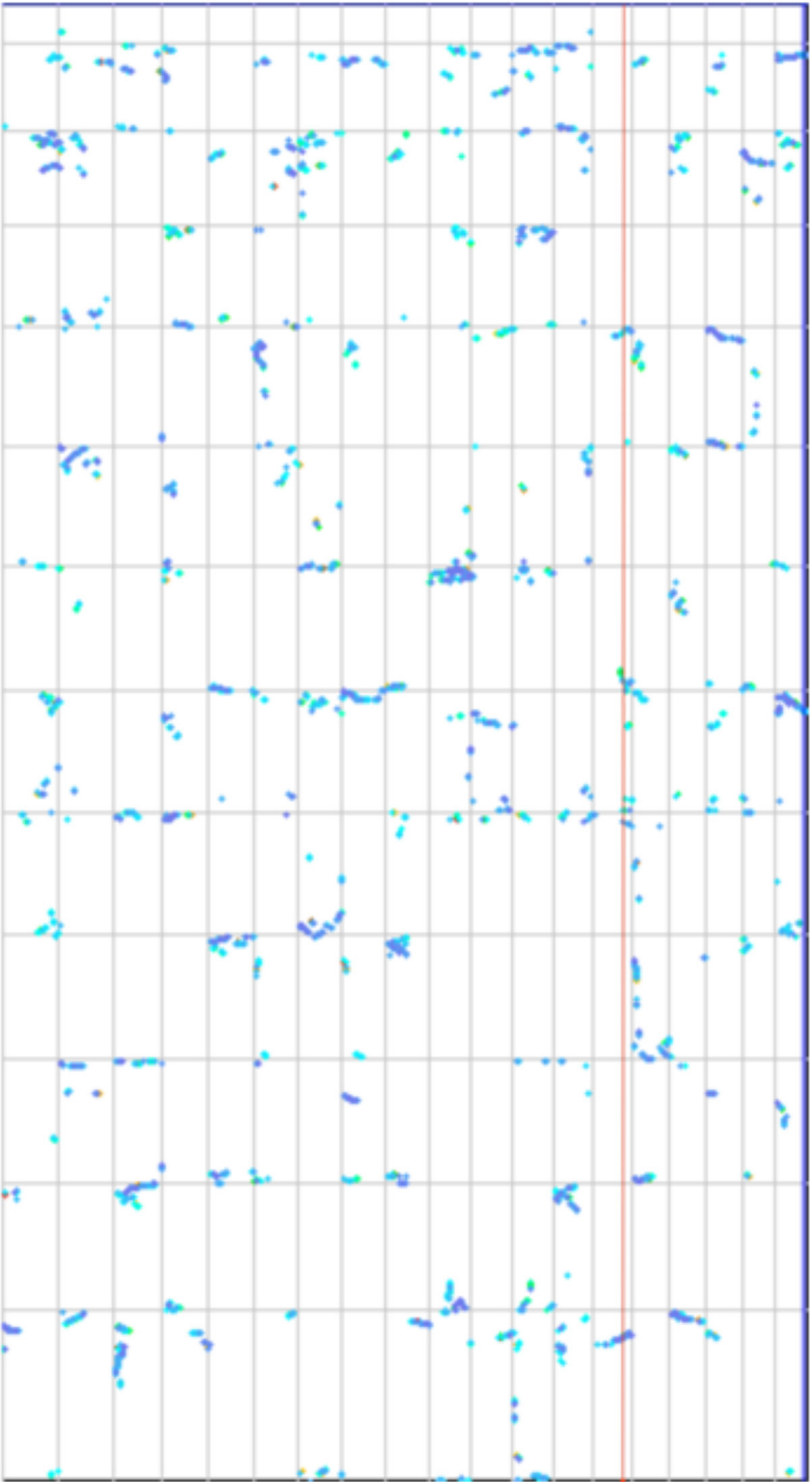
**Dotplot of syntenic blocks in a tomato-grape comparison**. Relatively dense distribution of syntenic blocks reflects necessity to conserve one gene of a duplicate pair when paralogy is lost and hence retain one orthologous pair. Vertical red line indicates four overlapping syntenic bloc

Figures 1 and 2 contrast the small number of paralogs within the tomato genome with the large numbers of orthologs between tomato and grape. The CoGe [2,1] function SynMap produced these two plots using the same set of parameters for assuring the duplicate genes identified have remained in the same syntenic context.

**Figure 3:**
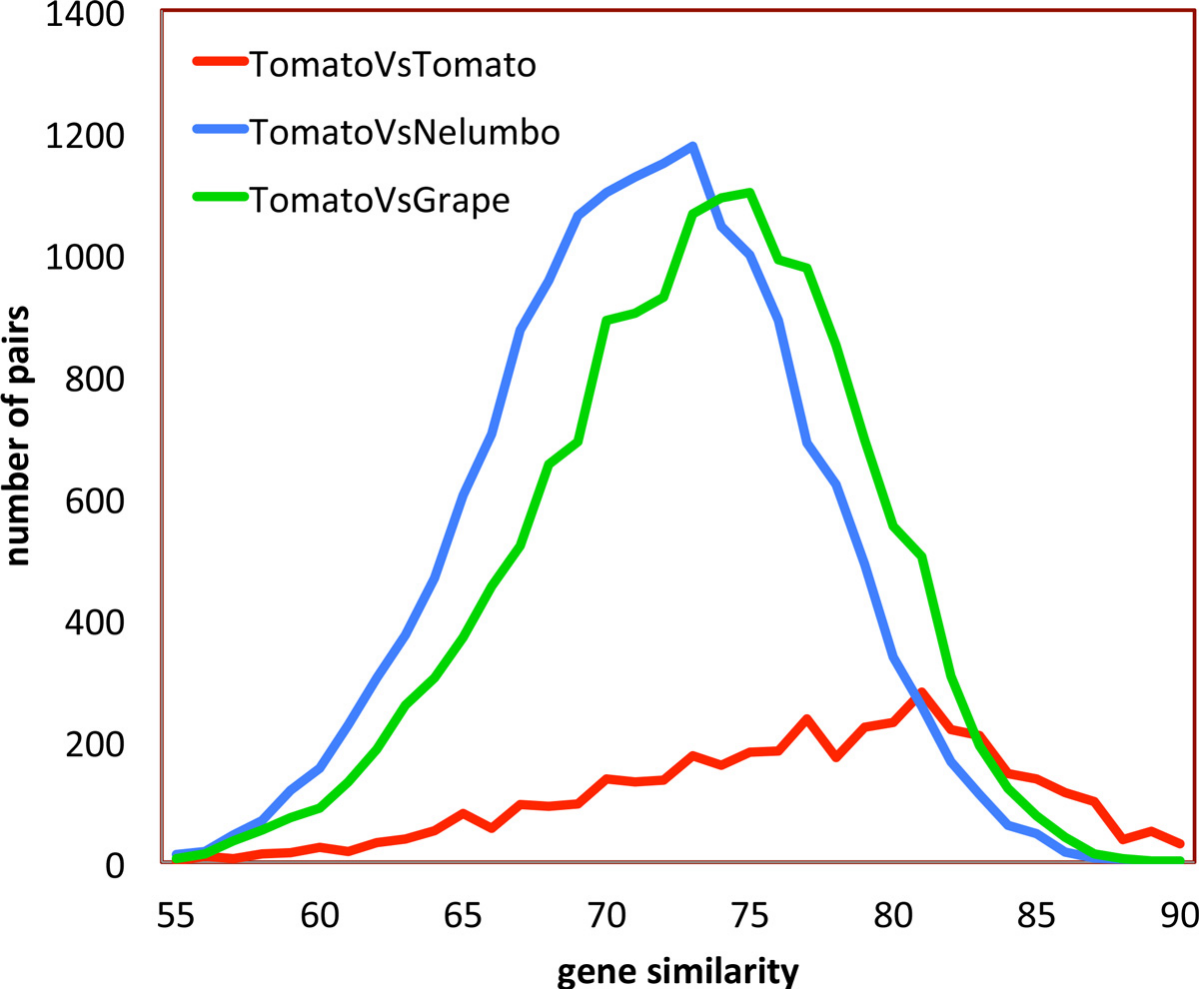
**Distribution of similarities between gene pairs**. *Nelumbo*-tomato divergence is earlier than grape-tomato divergence. Tomato paralogs reflect a mixture of two distributions, one the result of a relatively recent triplication.

By comparing the similarity of the genes in the pairs, we get a histogram whose mean reflects the age of the WGD event or the speciation event separating paralogs and orthologs, respectively. Figure 3 shows more precisely the very much larger number of orthologs than paralogs. This despite the fact that the bulk of the tomato paralogs originate in a more recent triplication (mean similarity 81 %) than the common triplication shared by tomato and grape (mean similarity 73 %).

That there is a recent tomato WGD or triplication is clear in Figure 3 from the single peak, but whether it is a duplication, triplication, or other event is not clear without further analysis of the genome [9,10]. The earlier triplication, well-known from the study of other core eudicots [11] is visible only as a skewness effect on the single-peaked distribution, which could be attributed to any number of early events.

## Overlapping blocks

The vertical red line in Figure 2 passes through four overlapping synteny blocks in four different tomato chromosomes. These blocks are orthologous with a single region on one grape chromosome. Examination of other regions of the grape genome show they form similar sets of multiple overlapping tomato synteny blocks. We use criteria of overlapping in a grape region spanning five or ten genes to recognize an overlap between two such synteny blocks. We denote the number of overlapping blocks spanning a contiguous region in grape the *multiplicity B *of this set of blocks, itself called a *superblock*. There is little difference in the results between five-and ten-gene overlaps, but increasing the required overlap beyond ten seriously impedes the identification of superblocks with high multiplicities, and decreasing it below five results superblocks of artifactually high multiplicities. In this analysis, we call grape the *reference *genome and tomato the *target *genome.

Let *f *(*B*) represent the empirical distribution function of block multiplicities over all superblocks. We can expect the support of *f *to include higher values of *B *for target genomes having undergone more WGD and having undergone triplications rather than duplications. However, the variable *B *is under downward pressure from processes such as fractionation (loss of duplicates due to dosage compensation) and rearrangement (which breaks up blocks by moving a part elsewhere in the genome).

## Fitting the distribution of multiplicities

**Figure 4:**
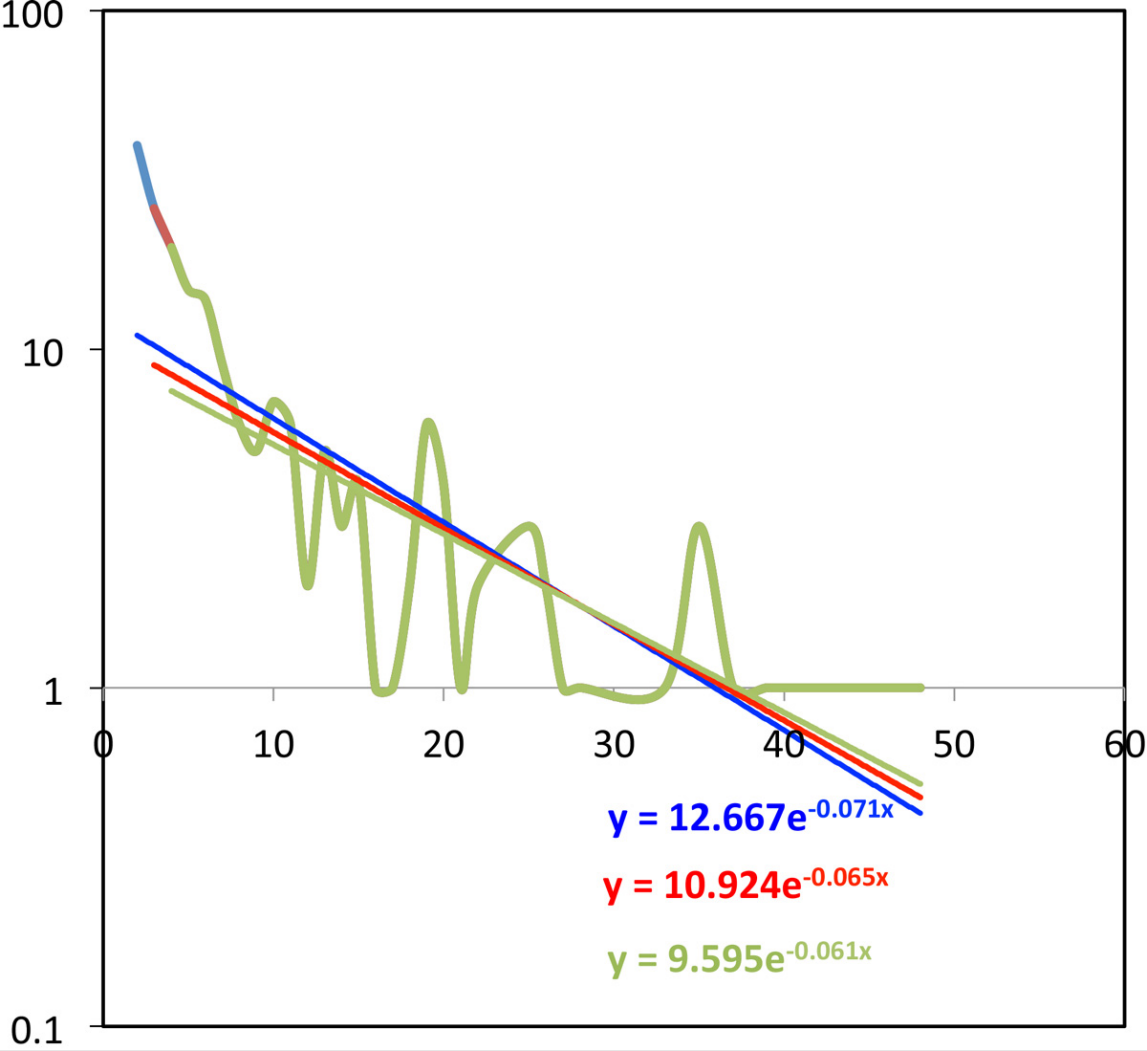
**Fitting the tail of *f*(*B*) with an exponential**. Green, red and blue curves include only *B ≥ *4, *B ≥ *3 and *B ≥ *2 (all *B*), respectively. For visibility, where *f *= 0, this is plotted as *f *= 1, but these points are not used in the fitting process.

The distribution *f *(*B*) is also affected by the recency of the latest event and, indeed, by the entire history of WGD events and the temporal spacing between them. The appearance of the distribution is exemplified in Figure 4, where grape is the reference and poplar is the target. As in this graph, the paucity of the data for higher values of *B *does not let us easily identify the shape of *f *. It is typical of the many such graphs, however, that the values of *f *(2) and *f *(3) are higher than an exponential fit to all the data would predict, and are very dependent on both the target and reference genomes. Thus we will use three different exponentials in the analysis that follows, one where *B ≥ *4, another where *B ≥ *3 and lastly one for all *B*, i.e., *B ≥ *2. The first gives higher weight to the tail of *f *in fitting the exponential than the other two, the second takes account of *B *= 3, while the third fits the entire domain of *B*.

In Figure 4, we see that in the log-linear equation fit *f*(*B*) = *ae^-cB^*, the estimates for the parameter *c *is more stable than for *a *when we change the definition of the tail of the distribution, and we confirmed that this observation holds for almost all pairs of target and reference genome. We retain only the parameter *c*, then, as a descriptor of the distribution.

## The effect of the reference genome

Figure 3 also shows that comparisons between tomato (the target genome) and the two reference genomes, grape and *Nelumbo*, do not produce coinciding histograms, showing that the latter was an earlier divergence, while the two core eudicots, tomato and grape are the products of a more recent divergence. In general, we find that the choice of reference genome tends to affect all the target-reference comparisons in a similar way.

## The CoGe platform

There are many programs designed to find synteny (or syntenic) blocks in the comparison of two or more eukaryotic gene orders, e.g., i-ADHoRe [12], DAGchainer [13], Cinteny[14], CYNTENATOR [15], MCScan [16] and DRIMM-Synteny [17]. These differ in search strategy, flexibility, performance and interpretation. SynMap is based on the DAGchainer algorithm, and balances sensitivity with rigour in finding blocks. It has the great advantage of immediate access to the vast genomic resources of CoGe organized in a common format and to the myriad of specially designed tools for analyzing and exploring the results of the comparative analysis.

## The credibility of syntenic blocks

An important parameter in SynMap we call "minL" the minimum number of homologs necessary to validate a syntenic block. Another parameter is the spacing allowed between genes in a block, but that is not directly relevant to our analysis. The default value of minL is 5, but minL = 4 and even minL = 3 also give credible results.

## Data and analysis

We used each one of the 15 genome in our sample as a reference genome for each of the other 14, repeating the analysis for minL = 3,4 and 5, and three definitions of the tail of *f *, for a total of 15 *× *14 *× *3 *× *3 = 1890 comparisons in all. For each reference genome, minL and tail cutoff, we compared the *c *for each pair of the 14 target genomes. For each such pair, there were thus 13 *× *3 *× *3 = 117 comparisons possible. For some minL, and some cutoffs, there was not enough data to calculate *c*, but around 100 comparisons were generally possible.

As a summary statistics, we simply counted for each pair of genomes, G and H, how many times *c*(*G*) was greater than *c*(*H*) and how many times it was less, out of 39 comparisons for each minL. The results for each minL were almost identical. We could thus have confidence that any biases due to reference genome, minL or cutoff would be neutralized.

We then ranked all 15 genomes according to how many other genomes they had larger (negative) *c*. The result was:

1 *Amborella trichopoda*

2 duckweed

3 rice

4 sorghum

5 *Nelumbo nucifer*

6 *Utricularia gibba*

7 clementine

8 coffee

9 grape

10 peach

11 poplar

12 *Mimulus guttatus*

13 cassava

14 *Arabidopsis thaliana*

15 tomato

**Figure 5:**
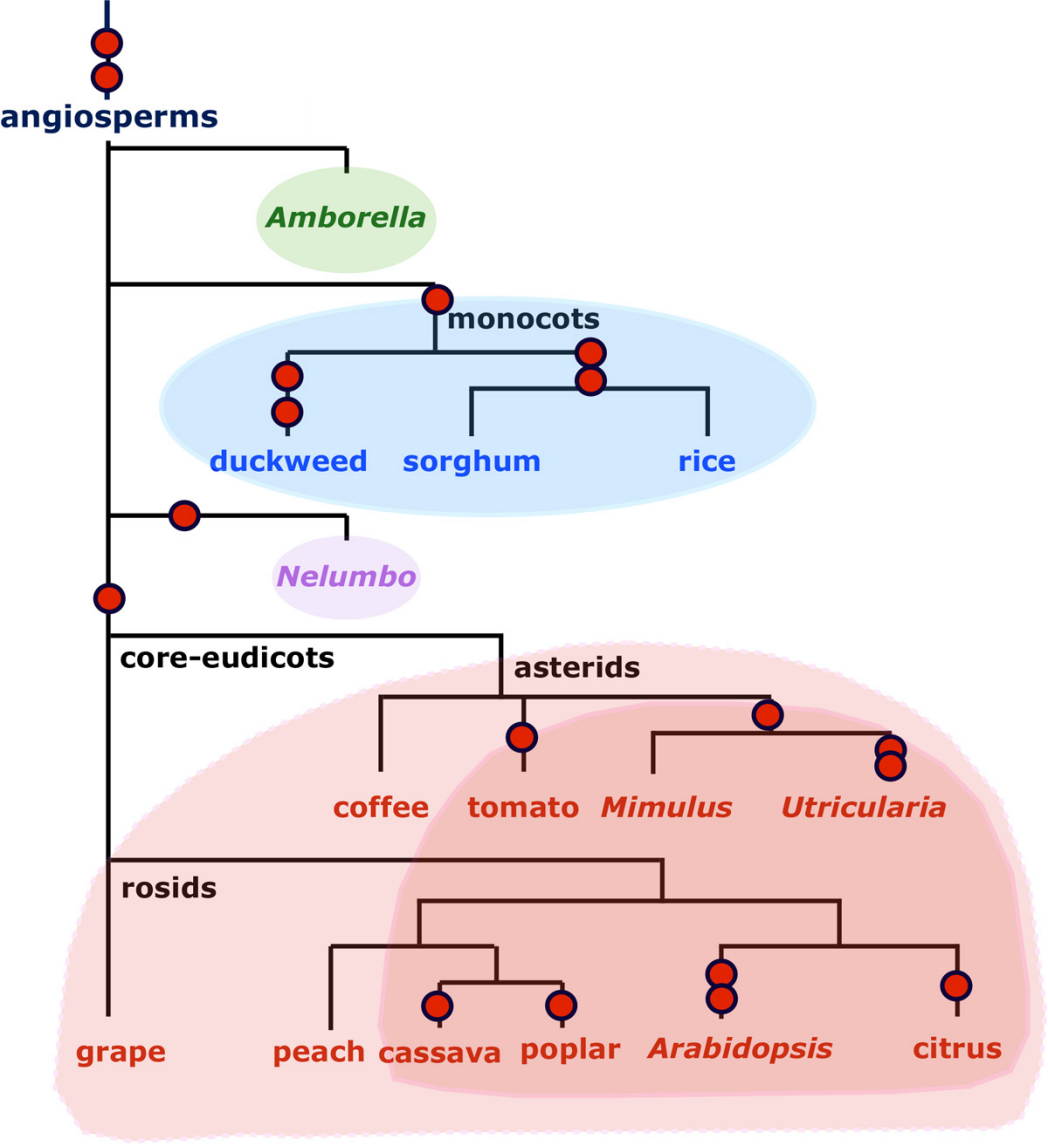
**The phylogenetic distribution of values of *c***. From top to bottom, we see decreasing values of *c*, reflecting a larger tail for *f*, according to the shaded groupings. Red circles indicate whole genome duplication events, including the core eudicot triplication and the tomato triplication.

In consulting Figure 5, the top of the list, the genomes with the largest *c *and hence the least persistent tail for *f *(*B*), was occupied by the basal angiosperm *Amborella *with no WGD since its divergence from the rest of the angiosperms, followed by the three monocots, followed by the stem eudicot *Nelumbo *with a single WGD, followed by the core eudicots having only the *γ *triplication, followed by those with more complex histories of doubling or tripling, where clementine and *Utricularia*, with their histories of additional duplication, are exceptions, being unexpectedly high on the list. The problem with clementine may be due to the quality of its assembly. For *Utricularia*, the explanation is its extremely rapid rate of fractionation [18].

## Conclusions

It is clear that the parameter *c *reflects the degree of WGD activity in the history of a genome. For example, within the core eudicots, the triplicated tomato and multiply doubled *Arabidopsis *have the lowest *c*, along with other recent WGD genomes, while the non-WGD genomes all have higher scores. *Nelumbo*, which has a WGD but not the core eudicot triplication, has a higher score and *Amborella *still higher. There is the notable exception of the monocots, which are known to have several WGD in their past. Indeed, in our sample of genomes, phylogenetic considerations can be invoked to explain the distribution of *c *as well as WGD history. Thus the next step will be to discriminate against these competing explanations by augmenting our analysis with further genomes as they become available, especially those more basal than the core eudicots.

## Competing interests

The authors declare that they have no competing interests.

## Authors' contributions

CZ and DS carried out most of the research and writing of this paper. DSM did much of the large scale data generation and participated in the preparation of the manuscript. VAA was instrumental in setting up the research problem and provided motivation and input for many of the analyses.
